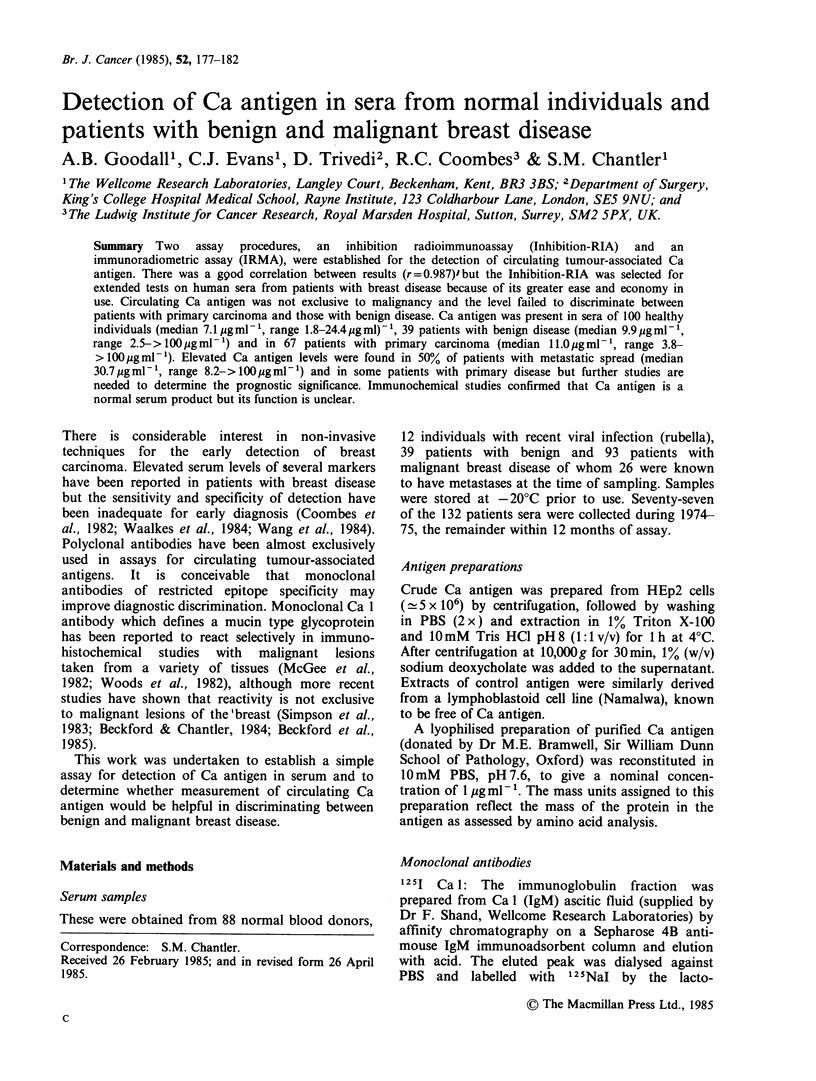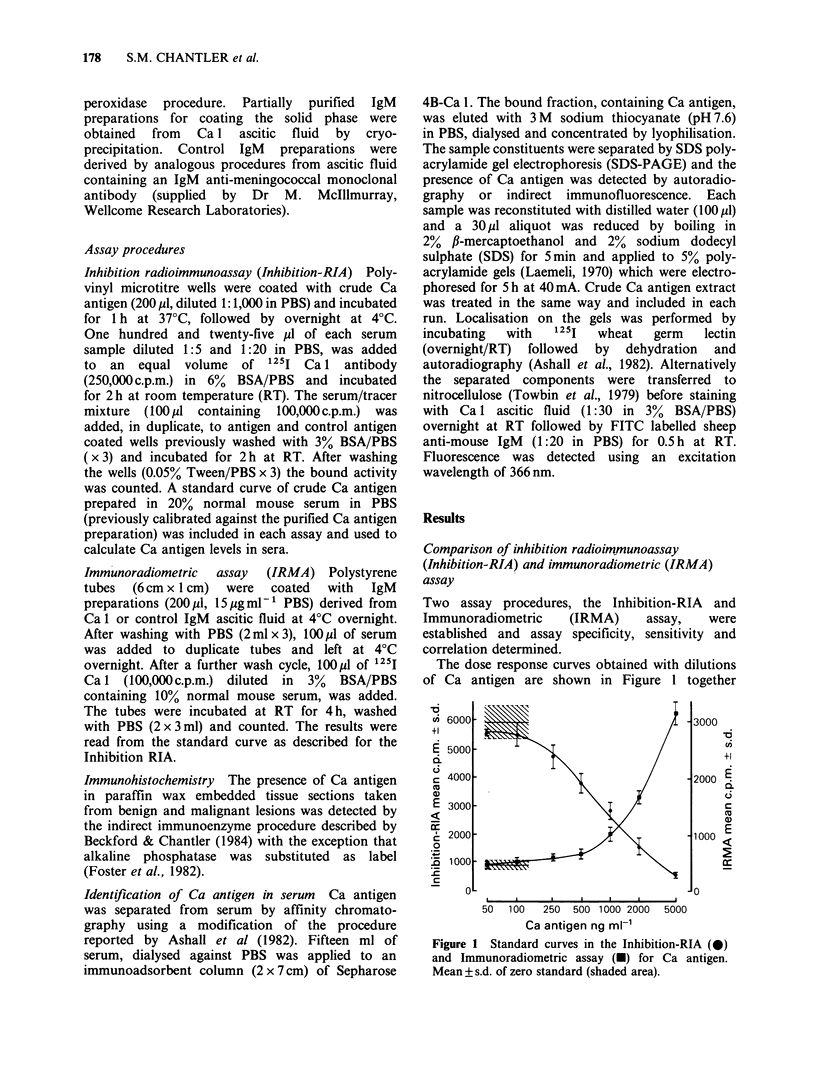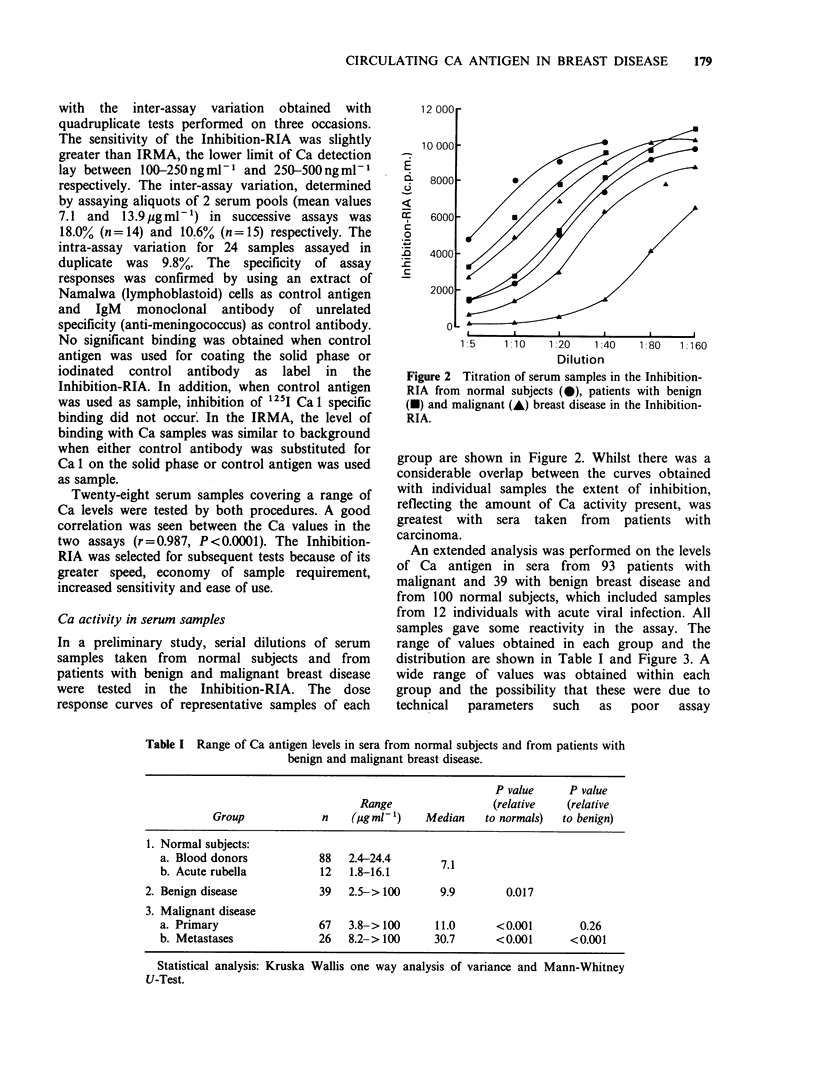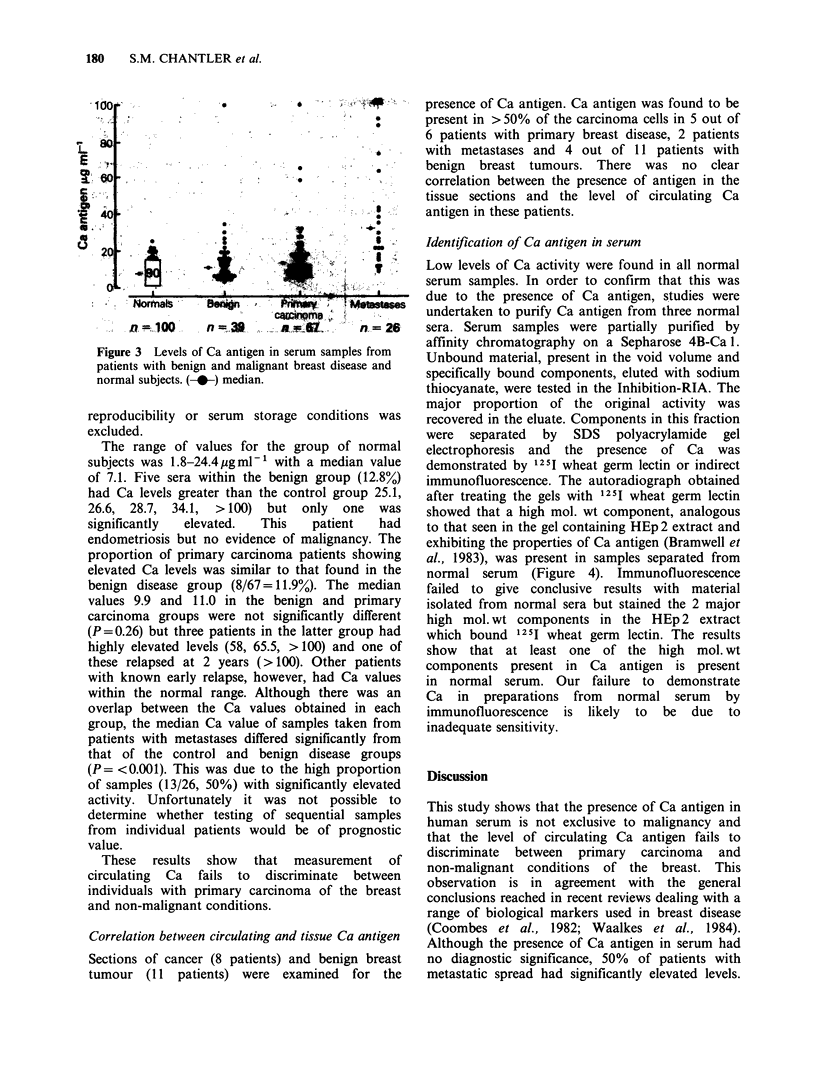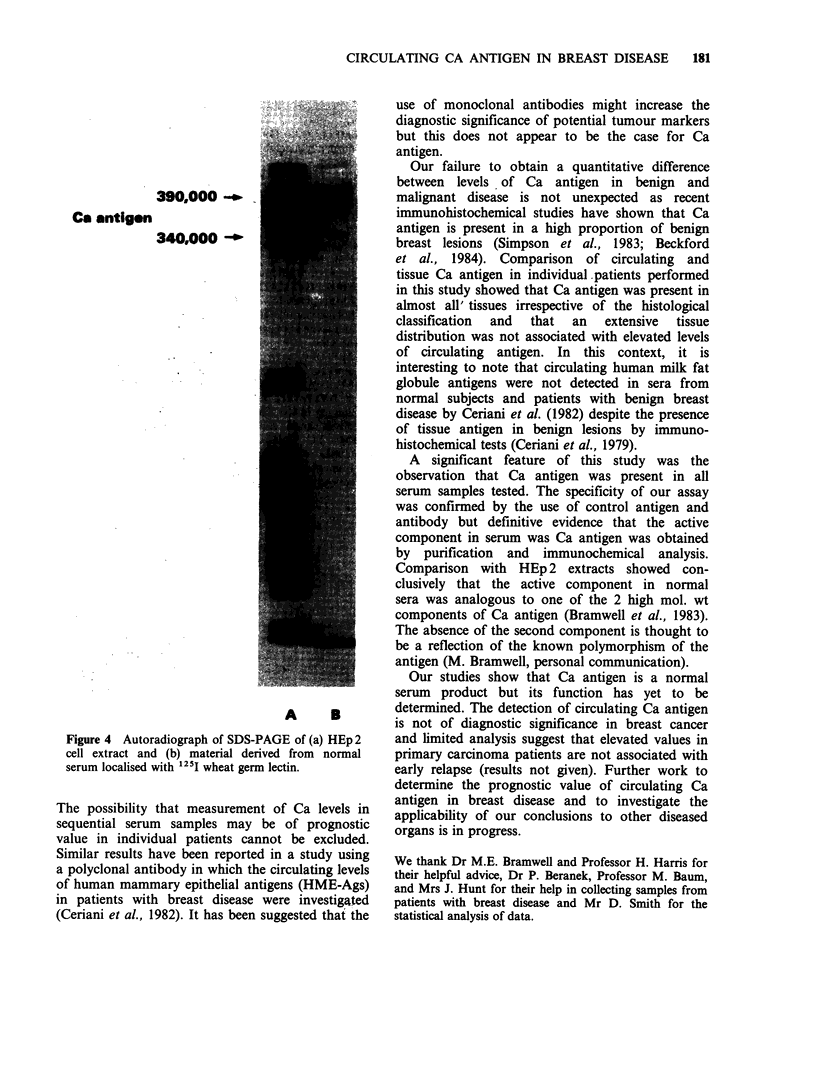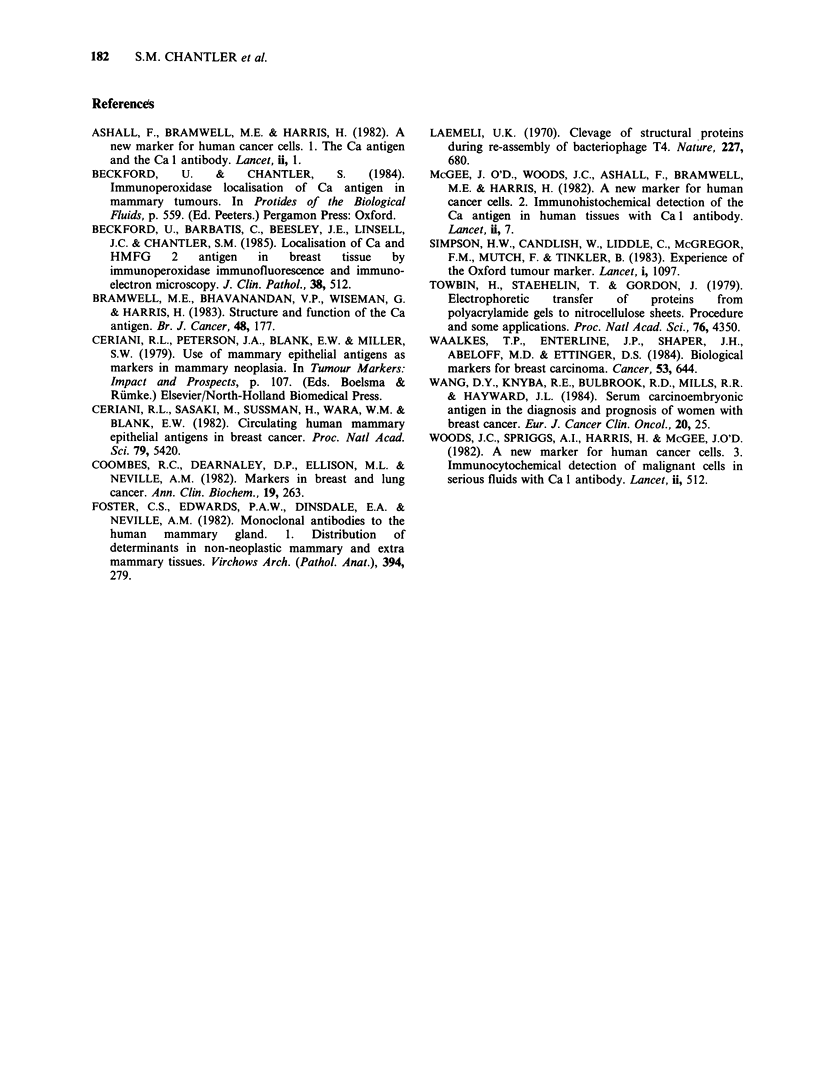# Detection of Ca antigen in sera from normal individuals and patients with benign and malignant breast disease.

**DOI:** 10.1038/bjc.1985.175

**Published:** 1985-08

**Authors:** A. B. Goodall, C. J. Evans, D. Trivedi, R. C. Coombes, S. M. Chantler

## Abstract

**Images:**


Br. J. Cancer (1985), 52, 177–182

### Detection of Ca antigen in sera from normal individuals and patients with benign and malignant breast disease

A.B. Goodall[1], C.J. Evans[1], D. Trivedi[2], R.C. Coombes[3] & S.M. Chantler[1]

[1]*The Wellcome Research Laboratories, Langley Court, Beckenham, Kent, BR3 3BS;* [2]*Department of Surgery, King's College Hospital Medical School, Rayne Institute, 123 Coldharbour Lane, London, SE5 9NU; and* [3]*The Ludwig Institute for Cancer Research, Royal Marsden Hospital, Sutton, Surrey, SM2 5PX, UK.*

**Summary** Two assay procedures, an inhibition radioimmunoassay (Inhibition-RIA) and an immunoradiometric assay (IRMA), were established for the detection of circulating tumour-associated Ca antigen. There was a good correlation between results ($r$=0.987) but the Inhibition-RIA was selected for extended tests on human sera from patients with breast disease because of its greater ease and economy in use. Circulating Ca antigen was not exclusive to malignancy and the level failed to discriminate between patients with primary carcinoma and those with benign disease. Ca antigen was present in sera of 100 healthy individuals (median 7.1 $\mu g\, ml^{-1}$, range 1.8–24.4 $\mu g\, ml)^{-1}$, 39 patients with benign disease (median 9.9 $\mu g\, ml^{-1}$, range 2.5–>100 $\mu g\, ml^{-1}$) and in 67 patients with primary carcinoma (median 11.0 $\mu g\, ml^{-1}$, range 3.8–>100 $\mu g\, ml^{-1}$). Elevated Ca antigen levels were found in 50% of patients with metastatic spread (median 30.7 $\mu g\, ml^{-1}$, range 8.2–>100 $\mu g\, ml^{-1}$) and in some patients with primary disease but further studies are needed to determine the prognostic significance. Immunochemical studies confirmed that Ca antigen is a normal serum product but its function is unclear.

There is considerable interest in non-invasive techniques for the early detection of breast carcinoma. Elevated serum levels of several markers have been reported in patients with breast disease but the sensitivity and specificity of detection have been inadequate for early diagnosis (Coombes *et al.*, 1982; Waalkes *et al.*, 1984; Wang *et al.*, 1984). Polyclonal antibodies have been almost exclusively used in assays for circulating tumour-associated antigens. It is conceivable that monoclonal antibodies of restricted epitope specificity may improve diagnostic discrimination. Monoclonal Ca 1 antibody which defines a mucin type glycoprotein has been reported to react selectively in immunohistochemical studies with malignant lesions taken from a variety of tissues (McGee *et al.*, 1982; Woods *et al.*, 1982), although more recent studies have shown that reactivity is not exclusive to malignant lesions of the breast (Simpson *et al.*, 1983; Beckford & Chantler, 1984; Beckford *et al.*, 1985).

This work was undertaken to establish a simple assay for detection of Ca antigen in serum and to determine whether measurement of circulating Ca antigen would be helpful in discriminating between benign and malignant breast disease.

### Materials and methods

### *Serum samples*

These were obtained from 88 normal blood donors, 12 individuals with recent viral infection (rubella), 39 patients with benign and 93 patients with malignant breast disease of whom 26 were known to have metastases at the time of sampling. Samples were stored at −20°C prior to use. Seventy-seven of the 132 patients sera were collected during 1974–75, the remainder within 12 months of assay.

### *Antigen preparations*

Crude Ca antigen was prepared from HEp2 cells ($\simeq 5\times10^6$) by centrifugation, followed by washing in PBS (2×) and extraction in 1% Triton X-100 and 10 mM Tris HCl pH 8 (1:1 v/v) for 1 h at 4°C. After centrifugation at 10,000 $g$ for 30 min, 1% (w/v) sodium deoxycholate was added to the supernatant. Extracts of control antigen were similarly derived from a lymphoblastoid cell line (Namalwa), known to be free of Ca antigen.

A lyophilised preparation of purified Ca antigen (donated by Dr M.E. Bramwell, Sir William Dunn School of Pathology, Oxford) was reconstituted in 10 mM PBS, pH 7.6, to give a nominal concentration of 1 $\mu g\, ml^{-1}$. The mass units assigned to this preparation reflect the mass of the protein in the antigen as assessed by amino acid analysis.

### *Monoclonal antibodies*

$^{125}I$ Ca 1: The immunoglobulin fraction was prepared from Ca 1 (IgM) ascitic fluid (supplied by Dr F. Shand, Wellcome Research Laboratories) by affinity chromatography on a Sepharose 4B anti-mouse IgM immunoadsorbent column and elution with acid. The eluted peak was dialysed against PBS and labelled with $^{125}$NaI by the lacto-

Correspondence: S.M. Chantler.
Received 26 February 1985; and in revised form 26 April 1985.

© The Macmillan Press Ltd., 1985

peroxidase procedure. Partially purified IgM preparations for coating the solid phase were obtained from Ca 1 ascitic fluid by cryoprecipitation. Control IgM preparations were derived by analogous procedures from ascitic fluid containing an IgM anti-meningococcal monoclonal antibody (supplied by Dr M. McIllmurray, Wellcome Research Laboratories).

### *Assay procedures*

*Inhibition radioimmunoassay (Inhibition-RIA)* Polyvinyl microtitre wells were coated with crude Ca antigen (200 μl, diluted 1:1,000 in PBS) and incubated for 1 h at 37°C, followed by overnight at 4°C. One hundred and twenty-five μl of each serum sample diluted 1:5 and 1:20 in PBS, was added to an equal volume of $^{125}$I Ca 1 antibody (250,000 c.p.m.) in 6% BSA/PBS and incubated for 2 h at room temperature (RT). The serum/tracer mixture (100 μl containing 100,000 c.p.m.) was added, in duplicate, to antigen and control antigen coated wells previously washed with 3% BSA/PBS (×3) and incubated for 2 h at RT. After washing the wells (0.05% Tween/PBS×3) the bound activity was counted. A standard curve of crude Ca antigen prepared in 20% normal mouse serum in PBS (previously calibrated against the purified Ca antigen preparation) was included in each assay and used to calculate Ca antigen levels in sera.

*Immunoradiometric assay (IRMA)* Polystyrene tubes (6 cm × 1 cm) were coated with IgM preparations (200 μl, 15 μg ml$^{-1}$ PBS) derived from Ca 1 or control IgM ascitic fluid at 4°C overnight. After washing with PBS (2 ml × 3), 100 μl of serum was added to duplicate tubes and left at 4°C overnight. After a further wash cycle, 100 μl of $^{125}$I Ca 1 (100,000 c.p.m.) diluted in 3% BSA/PBS containing 10% normal mouse serum, was added. The tubes were incubated at RT for 4 h, washed with PBS (2 × 3 ml) and counted. The results were read from the standard curve as described for the Inhibition RIA.

*Immunohistochemistry* The presence of Ca antigen in paraffin wax embedded tissue sections taken from benign and malignant lesions was detected by the indirect immunoenzyme procedure described by Beckford & Chantler (1984) with the exception that alkaline phosphatase was substituted as label (Foster *et al.*, 1982).

*Identification of Ca antigen in serum* Ca antigen was separated from serum by affinity chromatography using a modification of the procedure reported by Ashall *et al* (1982). Fifteen ml of serum, dialysed against PBS was applied to an immunoadsorbent column (2 × 7 cm) of Sepharose 4B-Ca 1. The bound fraction, containing Ca antigen, was eluted with 3 M sodium thiocyanate (pH 7.6) in PBS, dialysed and concentrated by lyophilisation. The sample constituents were separated by SDS polyacrylamide gel electrophoresis (SDS-PAGE) and the presence of Ca antigen was detected by autoradiography or indirect immunofluorescence. Each sample was reconstituted with distilled water (100 μl) and a 30 μl aliquot was reduced by boiling in 2% β-mercaptoethanol and 2% sodium dodecyl sulphate (SDS) for 5 min and applied to 5% polyacrylamide gels (Laemeli, 1970) which were electrophoresed for 5 h at 40 mA. Crude Ca antigen extract was treated in the same way and included in each run. Localisation on the gels was performed by incubating with $^{125}$I wheat germ lectin (overnight/RT) followed by dehydration and autoradiography (Ashall *et al.*, 1982). Alternatively the separated components were transferred to nitrocellulose (Towbin *et al.*, 1979) before staining with Ca 1 ascitic fluid (1:30 in 3% BSA/PBS) overnight at RT followed by FITC labelled sheep anti-mouse IgM (1:20 in PBS) for 0.5 h at RT. Fluorescence was detected using an excitation wavelength of 366 nm.

### Results

### *Comparison of inhibition radioimmunoassay (Inhibition-RIA) and immunoradiometric (IRMA) assay*

Two assay procedures, the Inhibition-RIA and Immunoradiometric (IRMA) assay, were established and assay specificity, sensitivity and correlation determined.

The dose response curves obtained with dilutions of Ca antigen are shown in Figure 1 together

**Figure 1** Standard curves in the Inhibition-RIA (●) and Immunoradiometric assay (■) for Ca antigen. Mean ± s.d. of zero standard (shaded area).

with the inter-assay variation obtained with quadruplicate tests performed on three occasions. The sensitivity of the Inhibition-RIA was slightly greater than IRMA, the lower limit of Ca detection lay between 100–250 ng ml$^{-1}$ and 250–500 ng ml$^{-1}$ respectively. The inter-assay variation, determined by assaying aliquots of 2 serum pools (mean values 7.1 and 13.9 $\mu$g ml$^{-1}$) in successive assays was 18.0% ($n=14$) and 10.6% ($n=15$) respectively. The intra-assay variation for 24 samples assayed in duplicate was 9.8%. The specificity of assay responses was confirmed by using an extract of Namalwa (lymphoblastoid) cells as control antigen and IgM monoclonal antibody of unrelated specificity (anti-meningococcus) as control antibody. No significant binding was obtained when control antigen was used for coating the solid phase or iodinated control antibody as label in the Inhibition-RIA. In addition, when control antigen was used as sample, inhibition of $^{125}$I Ca 1 specific binding did not occur. In the IRMA, the level of binding with Ca samples was similar to background when either control antibody was substituted for Ca 1 on the solid phase or control antigen was used as sample.

Twenty-eight serum samples covering a range of Ca levels were tested by both procedures. A good correlation was seen between the Ca values in the two assays ($r=0.987$, $P<0.0001$). The Inhibition-RIA was selected for subsequent tests because of its greater speed, economy of sample requirement, increased sensitivity and ease of use.

*Ca activity in serum samples*

In a preliminary study, serial dilutions of serum samples taken from normal subjects and from patients with benign and malignant breast disease were tested in the Inhibition-RIA. The dose response curves of representative samples of each group are shown in Figure 2. Whilst there was a considerable overlap between the curves obtained with individual samples the extent of inhibition, reflecting the amount of Ca activity present, was greatest with sera taken from patients with carcinoma.

**Figure 2** Titration of serum samples in the Inhibition-RIA from normal subjects (●), patients with benign (■) and malignant (▲) breast disease in the Inhibition-RIA.

An extended analysis was performed on the levels of Ca antigen in sera from 93 patients with malignant and 39 with benign breast disease and from 100 normal subjects, which included samples from 12 individuals with acute viral infection. All samples gave some reactivity in the assay. The range of values obtained in each group and the distribution are shown in Table I and Figure 3. A wide range of values was obtained within each group and the possibility that these were due to technical parameters such as poor assay

**Table I** Range of Ca antigen levels in sera from normal subjects and from patients with benign and malignant breast disease.

| *Group* | *n* | *Range ($\mu$g ml$^{-1}$)* | *Median* | *P value (relative to normals)* | *P value (relative to benign)* |
|---|---|---|---|---|---|
| 1. Normal subjects: | | | | | |
| a. Blood donors | 88 | 2.4–24.4 | 7.1 | | |
| b. Acute rubella | 12 | 1.8–16.1 | | | |
| 2. Benign disease | 39 | 2.5–>100 | 9.9 | 0.017 | |
| 3. Malignant disease | | | | | |
| a. Primary | 67 | 3.8–>100 | 11.0 | <0.001 | 0.26 |
| b. Metastases | 26 | 8.2–>100 | 30.7 | <0.001 | <0.001 |

Statistical analysis: Kruska Wallis one way analysis of variance and Mann-Whitney *U*-Test.

**Figure 3** Levels of Ca antigen in serum samples from patients with benign and malignant breast disease and normal subjects. (–●–) median.

reproducibility or serum storage conditions was excluded.

The range of values for the group of normal subjects was 1.8–24.4 $\mu g\, ml^{-1}$ with a median value of 7.1. Five sera within the benign group (12.8%) had Ca levels greater than the control group 25.1, 26.6, 28.7, 34.1, >100) but only one was significantly elevated. This patient had endometriosis but no evidence of malignancy. The proportion of primary carcinoma patients showing elevated Ca levels was similar to that found in the benign disease group (8/67 = 11.9%). The median values 9.9 and 11.0 in the benign and primary carcinoma groups were not significantly different ($P = 0.26$) but three patients in the latter group had highly elevated levels (58, 65.5, >100) and one of these relapsed at 2 years (>100). Other patients with known early relapse, however, had Ca values within the normal range. Although there was an overlap between the Ca values obtained in each group, the median Ca value of samples taken from patients with metastases differed significantly from that of the control and benign disease groups ($P = <0.001$). This was due to the high proportion of samples (13/26, 50%) with significantly elevated activity. Unfortunately it was not possible to determine whether testing of sequential samples from individual patients would be of prognostic value.

These results show that measurement of circulating Ca fails to discriminate between individuals with primary carcinoma of the breast and non-malignant conditions.

*Correlation between circulating and tissue Ca antigen*

Sections of cancer (8 patients) and benign breast tumour (11 patients) were examined for the presence of Ca antigen. Ca antigen was found to be present in >50% of the carcinoma cells in 5 out of 6 patients with primary breast disease, 2 patients with metastases and 4 out of 11 patients with benign breast tumours. There was no clear correlation between the presence of antigen in the tissue sections and the level of circulating Ca antigen in these patients.

*Identification of Ca antigen in serum*

Low levels of Ca activity were found in all normal serum samples. In order to confirm that this was due to the presence of Ca antigen, studies were undertaken to purify Ca antigen from three normal sera. Serum samples were partially purified by affinity chromatography on a Sepharose 4B-Ca 1. Unbound material, present in the void volume and specifically bound components, eluted with sodium thiocyanate, were tested in the Inhibition-RIA. The major proportion of the original activity was recovered in the eluate. Components in this fraction were separated by SDS polyacrylamide gel electrophoresis and the presence of Ca was demonstrated by $^{125}I$ wheat germ lectin or indirect immunofluorescence. The autoradiograph obtained after treating the gels with $^{125}I$ wheat germ lectin showed that a high mol. wt component, analogous to that seen in the gel containing HEp 2 extract and exhibiting the properties of Ca antigen (Bramwell *et al.*, 1983), was present in samples separated from normal serum (Figure 4). Immunofluorescence failed to give conclusive results with material isolated from normal sera but stained the 2 major high mol. wt components in the HEp 2 extract which bound $^{125}I$ wheat germ lectin. The results show that at least one of the high mol. wt components present in Ca antigen is present in normal serum. Our failure to demonstrate Ca in preparations from normal serum by immunofluorescence is likely to be due to inadequate sensitivity.

### Discussion

This study shows that the presence of Ca antigen in human serum is not exclusive to malignancy and that the level of circulating Ca antigen fails to discriminate between primary carcinoma and non-malignant conditions of the breast. This observation is in agreement with the general conclusions reached in recent reviews dealing with a range of biological markers used in breast disease (Coombes *et al.*, 1982; Waalkes *et al.*, 1984). Although the presence of Ca antigen in serum had no diagnostic significance, 50% of patients with metastatic spread had significantly elevated levels.

**Figure 4** Autoradiograph of SDS-PAGE of (a) HEp2 cell extract and (b) material derived from normal serum localised with $^{125}I$ wheat germ lectin.

The possibility that measurement of Ca levels in sequential serum samples may be of prognostic value in individual patients cannot be excluded. Similar results have been reported in a study using a polyclonal antibody in which the circulating levels of human mammary epithelial antigens (HME-Ags) in patients with breast disease were investigated (Ceriani *et al.*, 1982). It has been suggested that the use of monoclonal antibodies might increase the diagnostic significance of potential tumour markers but this does not appear to be the case for Ca antigen.

Our failure to obtain a quantitative difference between levels of Ca antigen in benign and malignant disease is not unexpected as recent immunohistochemical studies have shown that Ca antigen is present in a high proportion of benign breast lesions (Simpson *et al.*, 1983; Beckford *et al.*, 1984). Comparison of circulating and tissue Ca antigen in individual patients performed in this study showed that Ca antigen was present in almost all tissues irrespective of the histological classification and that an extensive tissue distribution was not associated with elevated levels of circulating antigen. In this context, it is interesting to note that circulating human milk fat globule antigens were not detected in sera from normal subjects and patients with benign breast disease by Ceriani *et al.* (1982) despite the presence of tissue antigen in benign lesions by immunohistochemical tests (Ceriani *et al.*, 1979).

A significant feature of this study was the observation that Ca antigen was present in all serum samples tested. The specificity of our assay was confirmed by the use of control antigen and antibody but definitive evidence that the active component in serum was Ca antigen was obtained by purification and immunochemical analysis. Comparison with HEp2 extracts showed conclusively that the active component in normal sera was analogous to one of the 2 high mol. wt components of Ca antigen (Bramwell *et al.*, 1983). The absence of the second component is thought to be a reflection of the known polymorphism of the antigen (M. Bramwell, personal communication).

Our studies show that Ca antigen is a normal serum product but its function has yet to be determined. The detection of circulating Ca antigen is not of diagnostic significance in breast cancer and limited analysis suggest that elevated values in primary carcinoma patients are not associated with early relapse (results not given). Further work to determine the prognostic value of circulating Ca antigen in breast disease and to investigate the applicability of our conclusions to other diseased organs is in progress.

We thank Dr M.E. Bramwell and Professor H. Harris for their helpful advice, Dr P. Beranek, Professor M. Baum, and Mrs J. Hunt for their help in collecting samples from patients with breast disease and Mr D. Smith for the statistical analysis of data.

### References

ASHALL, F., BRAMWELL, M.E. & HARRIS, H. (1982). A new marker for human cancer cells. 1. The Ca antigen and the Ca 1 antibody. *Lancet*, **ii**, 1.

BECKFORD, U. & CHANTLER, S. (1984). Immunoperoxidase localisation of Ca antigen in mammary tumours. In *Protides of the Biological Fluids*, p. 559. (Ed. Peeters.) Pergamon Press: Oxford.

BECKFORD, U., BARBATIS, C., BEESLEY, J.E., LINSELL, J.C. & CHANTLER, S.M. (1985). Localisation of Ca and HMFG 2 antigen in breast tissue by immunoperoxidase immunofluorescence and immuno-electron microscopy. *J. Clin. Pathol.*, **38**, 512.

BRAMWELL, M.E., BHAVANANDAN, V.P., WISEMAN, G. & HARRIS, H. (1983). Structure and function of the Ca antigen. *Br. J. Cancer*, **48**, 177.

CERIANI, R.L., PETERSON, J.A., BLANK, E.W. & MILLER, S.W. (1979). Use of mammary epithelial antigens as markers in mammary neoplasia. In *Tumour Markers: Impact and Prospects*, p. 107. (Eds. Boelsma & Rümke.) Elsevier/North-Holland Biomedical Press.

CERIANI, R.L., SASAKI, M., SUSSMAN, H., WARA, W.M. & BLANK, E.W. (1982). Circulating human mammary epithelial antigens in breast cancer. *Proc. Natl Acad. Sci.* **79**, 5420.

COOMBES, R.C., DEARNALEY, D.P., ELLISON, M.L. & NEVILLE, A.M. (1982). Markers in breast and lung cancer. *Ann. Clin. Biochem.*, **19**, 263.

FOSTER, C.S., EDWARDS, P.A.W., DINSDALE, E.A. & NEVILLE, A.M. (1982). Monoclonal antibodies to the human mammary gland. 1. Distribution of determinants in non-neoplastic mammary and extra mammary tissues. *Virchows Arch. (Pathol. Anat.)*, **394**, 279.

LAEMELI, U.K. (1970). Clevage of structural proteins during re-assembly of bacteriophage T4. *Nature*, **227**, 680.

McGEE, J. O'D., WOODS, J.C., ASHALL, F., BRAMWELL, M.E. & HARRIS, H. (1982). A new marker for human cancer cells. 2. Immunohistochemical detection of the Ca antigen in human tissues with Ca 1 antibody. *Lancet*, **ii**, 7.

SIMPSON, H.W., CANDLISH, W., LIDDLE, C., McGREGOR, F.M., MUTCH, F. & TINKLER, B. (1983). Experience of the Oxford tumour marker. *Lancet*, **i**, 1097.

TOWBIN, H., STAEHELIN, T. & GORDON, J. (1979). Electrophoretic transfer of proteins from polyacrylamide gels to nitrocellulose sheets. Procedure and some applications. *Proc. Natl Acad. Sci.*, **76**, 4350.

WAALKES, T.P., ENTERLINE, J.P., SHAPER, J.H., ABELOFF, M.D. & ETTINGER, D.S. (1984). Biological markers for breast carcinoma. *Cancer*, **53**, 644.

WANG, D.Y., KNYBA, R.E., BULBROOK, R.D., MILLS, R.R. & HAYWARD, J.L. (1984). Serum carcinoembryonic antigen in the diagnosis and prognosis of women with breast cancer. *Eur. J. Cancer Clin. Oncol.*, **20**, 25.

WOODS, J.C., SPRIGGS, A.I., HARRIS, H. & McGEE, J.O'D. (1982). A new marker for human cancer cells. 3. Immunocytochemical detection of malignant cells in serious fluids with Ca 1 antibody. *Lancet*, **ii**, 512.